# Implementing a perioperative efficiency initiative for orthopedic surgery instrumentation at an academic center: A comparative before-and-after study

**DOI:** 10.1097/MD.0000000000014338

**Published:** 2019-02-15

**Authors:** Richard Capra, Stefano A. Bini, Dawn E. Bowden, Katherine Etter, Matt Callahan, Richard T. Smith, Thomas Parker Vail

**Affiliations:** a University of California – San Francisco, San Francisco, CA; b Johnson & Johnson Medical Devices Companies, Somerville, NJ.

**Keywords:** arthroplasty, quality improvement, surgery, surgical instrumentation

## Abstract

Optimizing surgical instrumentation may contribute to value-based care, particularly in commonly performed procedures. We report our experience in implementing a perioperative efficiency program in 2 types of orthopedic surgery (primary total-knee arthroplasty, TKA, and total-hip arthroplasty, THA).

A comparative before-and-after study with 2 participating surgeons, each performing both THA and TKA, was conducted. Our objective was to evaluate the effect of surgical tray optimization on operating and processing time, cost, and waste associated with preparation, delivery, and staging of sterile surgical instruments. The study was designed as a prospective quality improvement initiative with pre- and postimplementation operational measures and a provider satisfaction survey.

A total of 96 procedures (38 preimplementation and 58 postimplementation) were assessed using time-stamped performance endpoints. The number and weight of trays and instruments processed were reduced substantially after the optimization intervention, particularly for TKA. Setup time was reduced by 23% (6 minutes, *P* = .01) for TKA procedures but did not differ for THA. The number of survey respondents was small, but satisfaction was high overall among personnel involved in implementation.

Optimizing instrumentation trays for orthopedic procedures yielded reduction in processing time and cost. Future research should evaluate patient outcomes and incremental/additive impact on institutional quality measures.

## 1 Introduction

Increasing efficiency for costly procedures is a strategic aim for health care systems around the world, and in 2015 the US Department of Health and Human Services announced a goal that 90% of Medicare fee-for-service payments be based on quality or value in the next 3 years.^[1]^ Knee and hip arthroplasty are the most commonly performed procedures in the United States when excluding childbirth and neonatal procedures, and demand is expected to grow strongly even under conservative assumptions.^[2,3]^ Value-based health care solutions can be applied in the perioperative setting to reduce overutilization of institutional resources while maintaining or improving patient outcomes.^[4]^ Streamlining the selection and configuration of surgical instrumentation is one promising component of value-based care. Audits of instrument usage across surgical settings have demonstrated that only 13% to 22% of instruments placed on surgical trays are used during a given operation.^[5]^ Reductions on the order of 25% to 50% are feasible, even allowing for infrequently used but necessary instruments requested by surgeons.^[6–8]^

Research on surgical tray efficiency in orthopedic surgery to date has mostly been limited to implementation of patient-specific instrumentation in total-knee arthroplasty (TKA). The purpose of the present study was to evaluate our experience implementing a perioperative efficiency program to optimize surgical instrumentation for a joint arthroplasty team already operating at high levels of efficiency. We aimed to address product-, cost-, and process-related variation while supporting patient outcomes and surgeon preferences.

## 2 Methods

We designed and implemented optimized instrument sets for patients undergoing primary TKA or total-hip arthroplasty (THA) at this institution. The study was conceived as a feasibility pilot, with a pre- and postevaluation of practice change. No randomized comparison was planned or implemented. No patient-identifying information was collected and standard protocols were followed for operating room (OR) accessibility. The study was conducted as part of the hospital's quality improvement initiative and was therefore considered exempt from Institutional Review Board review requirements. We followed current guidelines for quality improvement studies in preparing this report.^[9]^

### 2.1 Surgical setting

Patients undergoing primary TKA or THA procedures at the University of California - San Francisco (UCSF) Parnassus OR, with basic and noncomplicated joints (e.g., basic osteoarthritis cases), were considered eligible. Two surgeons participated, each of whom perform between 150 and 200 TKA and THA procedures per year. Both participating surgeons had extensive experience with the implant systems used in the study. The implant systems used throughout the pre- and postimplementation study periods came from a single vendor, Johnson & Johnson Medical Devices Companies. This consisted of one total-knee system (ATTUNE Knee System) for TKA, and one total-hip system (PINNACLE Hip Solutions, SUMMIT Tapered Hip System) for THA.

### 2.2 Intervention

**Figure 1:**
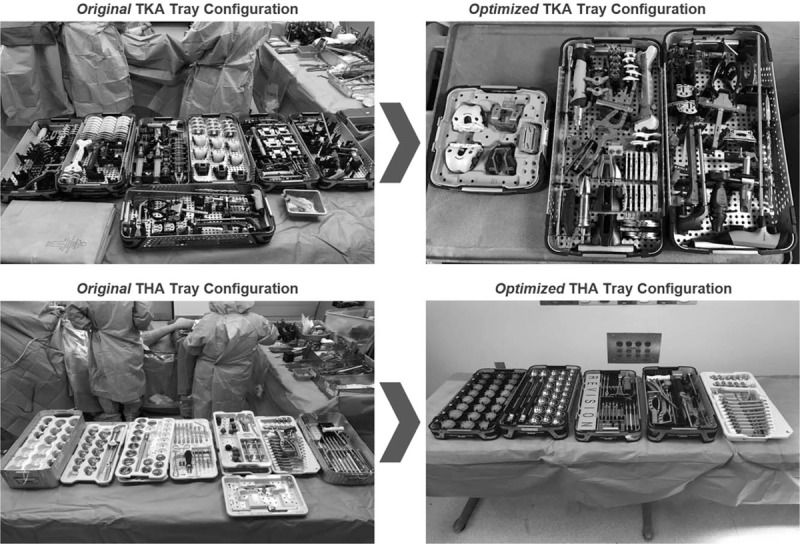
Optimized tray configuration. TKA = total-knee arthroplasty, THA = total-hip arthroplasty.

The intervention consisted of optimized, surgical instrumentation tray configurations for TKA and THA. The optimized tray configurations were designed to reduce the number of trays and instruments while maintaining consistent availability of equipment (Fig. 1). Tray configurations were customized for the 2 participating surgeons. Both surgeons utilized the same optimized tray configuration for THA, while each used a slightly different optimized configuration with the same number of instruments for TKA.

The new instrument configurations were planned in Q4 2016. Baseline data were collected in February 2017, including a preimplementation survey of surgeons, OR staff, and Sterile Processing Department personnel. The new tray configurations were deployed over the course of several months starting in March 2017, during which time procedure data were not collected to allow adjustment of processes before assessing the impact of the reconfigured trays. After this settle down/phase in period, postimplementation data were collected on all eligible procedures performed by participating surgeons in July and August 2017. A follow-up survey of personnel was conducted in September 2017.

### 2.3 Primary and secondary outcomes

The OR setup time was the primary endpoint for this study. Setup time was defined as the period between wheeling in the 1st instruments and placing all items on the back table in place for surgery. An in-room observer used tracking software to collect timestamped data on the duration of predefined steps in each procedure. The observer was trained on use of the software and the definitions of each timing variable, but was not blinded to the use of original vs optimized tray configurations, due to feasibility and the pre-post nature of the study.

Secondary endpoints included clean down time, total OR time, number of trays and instruments being processed, tray weight, number of trays in blue wrap vs rigid sterile containers, and cost estimates based on the observed parameters. Clean down time commenced when the 1st instrument was put away following definitive implantation and ended once the OR was clean and ready for the next procedure. In addition to the outcomes measured during procedures, provider and staff satisfaction with the intervention were assessed with the pre- and postsurveys mentioned earlier.

### 2.4 Analyses

The primary comparison of interest was between the pre- and postintervention periods. A sample size of 76 patients (38 prior to implementation and a minimum of 38 postintervention) was determined to be sufficient to detect a 5-minute reduction in the primary endpoint with 80% power. As we were only concerned with the potential for improvement for this effort to enhance efficiency, a 1-sided *t* test was used for the power calculation. The test of significance used to assess pre-post differences in outcomes was the Kolmogorov–Smirnov 2-sample test, which is a nonparametric test appropriate for use with data that are not normally distributed. Endpoints were assessed for all procedures (pre vs post) and were also stratified by type of surgery (knee vs hip). For continuous variables, means, medians, ranges, and standard deviations of the mean (standard deviation) were computed. The percentage change in the primary endpoint from pre- to postimplementation was calculated as the difference in means divided by the baseline value. *P*-values <.05 were regarded as statistically significant (no adjustment was made for multiple comparisons). Analyses were performed using SAS Enterprise Guide 7.1 (SAS Institute, Inc, Cary, NC).

## 3 Results

The study population consisted of 38 procedures during the preimplementation period and 58 during the postimplementation period, all of which were completed by the same 2 surgeons. Of the 96 procedures, nearly half (49%) were primary TKA and 51% were primary THA. Procedure characteristics were similar in the pre- and postintervention groups, except for a preponderance of cemented primary hip surgeries preimplementation, vs 97% cementless after the optimized trays were in use (Table 1). Patient characteristics, including indication for surgery, were not collected. Based on the differences in cemented vs cementless THA, more procedures in the preimplementation phase were performed for patients with hip fracture.

**Table 1:**
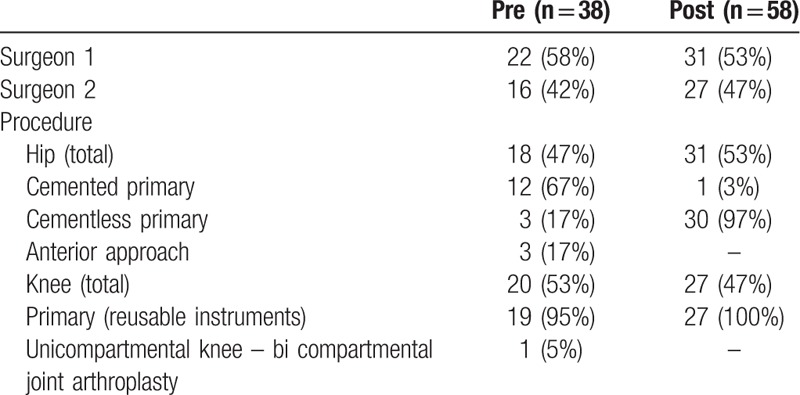
Procedure characteristics: pre- and postimplementation of tray efficiency protocol.

### 3.1 Tray optimization intervention

The 2 participating surgeons used comparable numbers of trays and instruments at baseline, while the tray optimization exercise greatly reduced the number of trays used during the postimplementation period. The original TKA tray configuration consisted of 214 instruments on 8 trays for surgeon 1 and 174 instruments on 8 trays for surgeon 2. The original THA configuration consisted of 14 trays (143 instruments) for both surgeons, many of which were seldom used. Many instruments in the trays were simply included as a result of historical tray construction without specific attention to the frequency of use. During the optimization exercise, the surgeons reached agreement on elimination of instruments that were infrequently used. Tray configuration strategies included placement of implant instruments in wire baskets inside of rigid sterile containers, paring down the number of existing original equipment manufacturer trays, and creating specialized made-to-order trays via 3rd-party suppliers (for TKA only at the time of this work).

In the postimplementation period, 3 trays were used for TKA and 6 for THA, a decrease of 62.5% and 57.1%, respectively. The number of instruments was reduced by 43.6% (TKA) and 17.5% (THA). There was a 47.4% reduction in total weight due to the optimized tray configurations (34% for hip instrumentation and 63.9% for knee instrumentation). The original tray configuration for TKA was 69.9 lbs. and the optimized set was 25.3 lbs; for THA, the original set was 86.3 lbs while the optimized set was 57.0 lbs.

### 3.2 Efficiency outcomes

**Figure 2:**
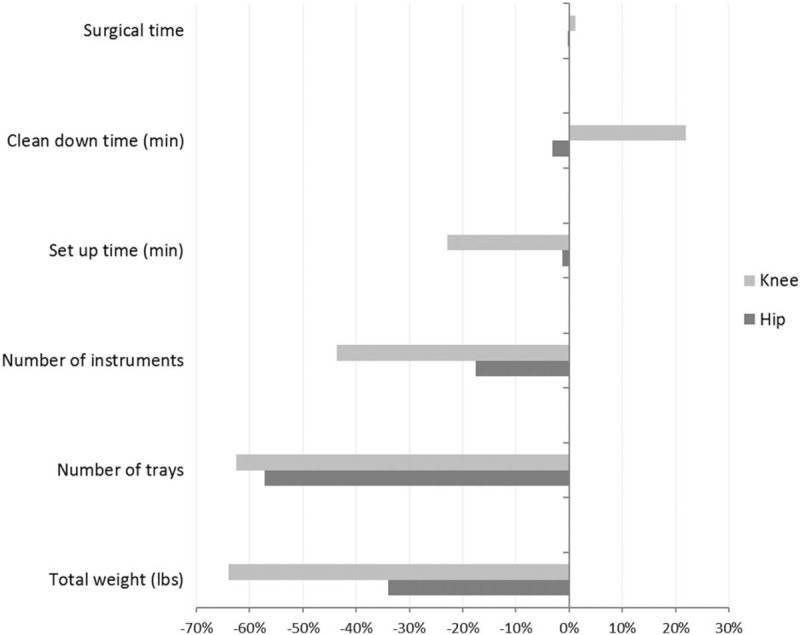
Percent change in study endpoints, ^∗^*P* = .06 for pre- vs postimplementation of optimized surgical trays, ^†^Reduction of at least 10% from pre- to postimplementation of optimized surgical trays. These endpoints are comparisons of the new instrument and tray configuration to the previous configuration (counts, not means with variation); no statistical test of significance is available.

Average setup time declined by 3 minutes across all procedures, a modest improvement which did not reach statistical significance (*P* = .06). Among TKA procedures, a 23% decrease (6-minute reduction) in setup time was observed, slightly higher than the 5 minutes assumed to be clinically significant in study planning (*P* = .01). Changes in clean down time (in aggregate, 2.4 minutes longer in the postimplementation group (*P* = .36), and total OR time were not significantly different after the intervention (Fig. 2). No differences in effect were seen when stratifying outcomes by surgeon. Multivariable analysis was not feasible due to the small number of baseline variables. When stratified by type of hip procedure (given the differences between pre- and postimplementation use of cement), average cementless THA setup time declined from 27.3 to 24.8 minutes, and clean down time from 51.7 to 31.5 minutes. However, only 3 preimplementation cementless cases were available for comparison.

Costs were estimated by multiplying the reduction in the number of trays by the number of primary joint arthroplasties per year (700) and the estimated cost of $75 to sterilize 1 tray.^[10]^ The optimized instrumentation configuration is estimated to provide an annual savings of $159,600 in sterile processing costs. Environmental impact is also reduced, due to less use of blue wrap, natural gas, electricity, and water. In addition, if decreased setup times can be maintained while stabilizing clean down time, projected savings of $99,000 would result from the reduction in total OR turnover time.^[11]^

When surveyed, surgeons and hospital staff reported positive feedback on the intervention. Prior to the intervention, 100% of 13 survey respondents expected the tray efficiency intervention would add value for the institution, and that a reduction in size, number, and/or weight of instrument trays could have a positive impact on patient care. After the intervention, only 5 individuals responded to a similar survey. Four of 5 agreed that the intervention had a positive impact on patient care, and that the program would add some or a good deal of value for other specialties (e.g., spine surgery). Sixty percent of respondents felt that the program offered additional value over other facility-led programs, and the same number (60%) agreed that the workload/time commitment required during program implementation was reasonable. Response rate for the survey was 62% preimplementation and 39% postimplementation.

## 4 Discussion

This study demonstrates the efficiency and cost benefit of streamlining surgical instrumentation trays in an academic medical center even when adopted by 2 surgeons in a 5-surgeon arthroplasty practice. As the optimization pertained to the instrumentation set of 1 implant manufacturer, the impact of the intervention could only be measured on the surgeons who routinely used that manufacturer's products. In the case of this particular institution, only 2 of the 5 staff surgeons could be included. The magnitude of reduction in the number of trays (57% for hip and 63% for knee) was in line with previously published estimates of instrument reduction in joint arthroplasty (43–66%), nearly all of which described patient-specific instrumentation vs conventional instrumentation in TKA (Table 2). Optimizing instrument inventory for surgery requires a high level of coordination and planning to ensure success. Lower tray and instrument counts could be achieved in the future with digital templating and consolidated shipments to support patient-specific tray and instrument sets. Future development and research should focus on integrated approaches to exchange data between health systems and their distribution centers to deliver advanced case management of both implants and instruments for orthopedic surgery.

**Table 2:**
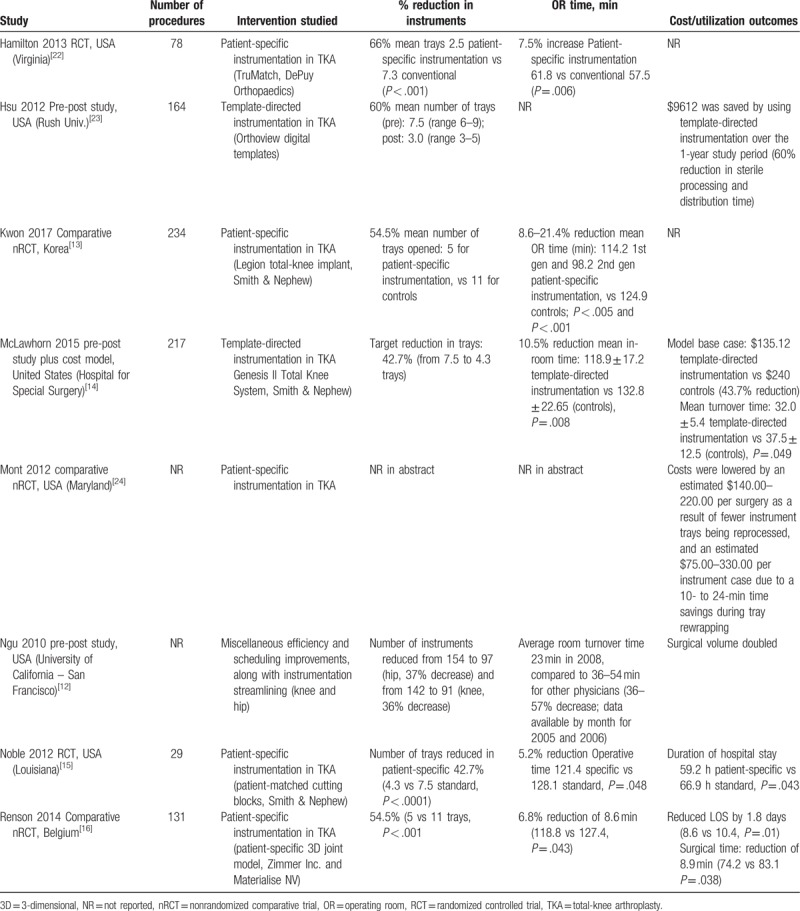
Prior studies of instrumentation reduction/streamlining tray configuration in joint arthroplasty.

The reduction in the number of instruments for THA was smaller than that observed for TKA. It should be noted that the TKA technique may be more conducive to reduced instrumentation, given that precise cuts are made once size is determined. Furthermore, with respect to bone preparation, only the instruments needed for the final cuts are necessary. In contrast, THAs involve “ream up” and “broach up” techniques for acetabular and femoral bone preparation. For THA, there was not a noticeable reduction in setup time, raising the possibility of a dosage effect or minimum threshold in terms of instrument reduction that needs to be reached to impact processes. We did not experience the reduction in total OR time which has been reported in some TKA studies.^[12–16]^ As the study progressed, we noted that both total OR time and clean down time included processes that we could not have affected by this intervention, including custodian cleanup of the OR. Other settings in which efforts to streamline surgical instrumentation have been evaluated include gynecologic surgery, robot-assisted prostatectomy, central venous catheter placement, and endovascular neurosurgical procedures.^[17–20]^ In general, these process changes have been accomplished without negative impact on patient outcomes or surgeon/staff satisfaction.

Further, the Sterile Processing Department noted additional project impacts, including the transition of all implant instrument sets to rigid sterile containers. This process change eliminates issues related to holes in blue wrap, greatly reduces the chance for contamination and surgical delay due to the need for reprocessing sets unexpectedly, and reduces space required in the Sterile Processing Department. The new tray configuration allows for storage of 2 complete systems in space that previously accommodated 1 complete system. Minimizing the loaner process is also an important objective for the department as it results in less time, processing, confusion, tracking issues, and logistical issues. With predictive analytics, the department anticipates stocking 2 more of each of the primary base trays and only 2 to 3 of the 6 femoral sizer trays. These stocking changes will potentially eliminate the need for loaner trays except for surgery days on which multiple surgeons in different rooms need the system. Overall, there are fewer instruments to process; this is associated with a faster assembly time and lower cost to complete the sterilization process, from decontamination through assembly, sterilization, and storage.

Strengths of this study include broad patient selection criteria, a standardized data collection instrument, and observation alongside routine care (rather than in an experimental, trial setting), which did not require substantial additional effort on the part of hospital staff. We used a settle down/phase in period while the optimized trays were 1st being used, to minimize potential bias from a learning curve for new processes. An effort was made to survey hospital staff impacted by the process change both before and after implementation.

Limitations of the study include the lack of a randomized control group, the small sample size especially with regard to the staff survey, and the absence of recorded clinical outcomes for TKA or THA. Survey responses, especially postimplementation, were limited due to staffing schedule and team size. While finding people to complete a survey preoperatively was relatively straight forward and could include any number of technicians and personnel, the people who initially responded to the survey may not have been associated with the optimized trays during the implementation phase on a consistent enough basis to be willing to offer an opinion. This is because of the size of the team: the orthopedic service runs more than 3 rooms per day and surgical team members scrub and support a number of subservices based on varying needs in the OR on any given day. Thus, on any given day a surgeon may have a completely different team. For future research, targeting the survey only to those staff members more likely to assist the surgeons involved in the project might be indicated though this may not actually be possible. A follow-up study is planned to evaluate patient-reported outcomes and institutional quality metrics. The unblinded nature of the pre- and postintervention observation process, while practical for collecting data, could have affected performance by virtue of staff awareness that they were being observed; however, this effect should have been similar in both the pre- and postintervention periods.

Our data come from a single academic institution, with self-selected and highly experienced surgeons participating in the intervention. Results may not be generalizable to other settings; in particular, prior institutional efforts to streamline surgical processes indicate that this institution was starting from a high baseline in terms of efficiency.^[12,21]^ Further studies with more surgeons, at a variety of experience levels, should be conducted. Additionally, future research should include an assessment of how frequently surgeons required additional instruments beyond what is included in the original order to complete the case. While, anecdotally, this was a very rare event, prospective data collection would strengthen the conclusions of future research. Even ambitious quality improvement initiatives can fail to achieve statistically significant improvement in patient outcomes.^[25]^ However, given the importance of perioperative care in the US health care system, there is a case to be made for continued attempts to implement evidence-based adjustments to usual surgical practice, while tracking the impact on patients. Even small increments of increased efficiency, for instance on OR transition times, may have impact when applied to a frequently performed procedure such as joint arthroplasty. In future research, patient-centered outcomes including safety, effectiveness, and quality of life impacts should be evaluated across a range of settings and contexts.

Larger sample sizes may be needed to pinpoint effects of incremental changes to complex processes, including potential impact on institution-wide quality metrics. Lastly, controlled studies with the assessor blinded to the group assignment, if feasible, would improve the reliability of this type of observational evidence.

We demonstrated that a surgical tray efficiency program can be successfully implemented for orthopedic procedures. The program reduced OR setup time, the number of trays and instruments, and costs associated with sterilization. Anecdotally, surgeon and staff satisfaction with the intervention was high.

## Author contributions

**Conceptualization:** Richard Capra, Stefano A. Bini, Dawn E. Bowden, Katherine Etter, Matt Callahan, Richard T. Smith, Thomas Parker Vail.

**Data curation:** Katherine Etter.

**Formal analysis:** Katherine Etter.

**Funding acquisition:** Richard Capra, Dawn E. Bowden.

**Investigation:** Richard Capra, Stefano A. Bini, Katherine Etter, Matt Callahan, Richard T. Smith, Thomas Parker Vail.

**Methodology:** Richard Capra, Stefano A. Bini, Dawn E. Bowden, Katherine Etter, Matt Callahan, Richard T. Smith, Thomas Parker Vail.

**Project administration:** Richard Capra, Dawn E. Bowden.

**Resources:** Richard Capra, Stefano A. Bini, Matt Callahan, Richard T. Smith, Thomas Parker Vail.

**Supervision:** Richard Capra, Dawn E. Bowden.

**Validation:** Richard Capra, Stefano A. Bini, Dawn E. Bowden, Richard T. Smith.

**Visualization:** Richard Capra, Dawn E. Bowden.

**Writing – original draft:** Dawn E. Bowden, Katherine Etter.

**Writing – review & editing:** Richard Capra, Stefano A. Bini, Dawn E. Bowden, Katherine Etter, Matt Callahan, Richard T. Smith, Thomas Parker Vail.
